# Energy audit and carbon footprint in trawl fisheries

**DOI:** 10.1038/s41597-022-01478-0

**Published:** 2022-07-20

**Authors:** Antonello Sala, Dimitrios Damalas, Lucio Labanchi, Jann Martinsohn, Fabrizio Moro, Rosaria Sabatella, Emilio Notti

**Affiliations:** 1 National Research Council, Institute of Marine Biological Resources and Biotechnologies (CNR-IRBIM), Ancona, Italy; 2 Institute of Marine Biological Resources and Inland Waters, Hellenic Centre for Marine Research, Heraklion, Greece; 3 MARBLY scarl, Salerno, Italy; 4 European Commission, Joint Research Centre (JRC), Ispra, VA Italy; 5 NISEA, Fisheries and Aquaculture Economic Research, Salerno, Italy

**Keywords:** Energy efficiency, Energy management, Environmental impact

## Abstract

The combustion of fossil fuels is considered a major cause of climate change, which is why the reduction of emissions has become a key goal of the Paris climate agreement. Coherent monitoring of the energy profile of fishing vessels through an energy audit can effectively identify sources of inefficiency, allowing for the deployment of well-informed and cost-efficient remedial interventions. We applied energy audits to a test fleet of ten vessels, representing three typical Mediterranean trawl fisheries: midwater pair trawl, bottom otter trawl, and Rapido beam trawl. Overall, these fisheries use approximately 2.9 litres of fuel per kilogram of landed fish, but the fuel consumption rate varies widely according to gear type and vessel size. This amount of fuel burned from capture to landing generates approximately 7.6 kg∙CO2/kg fish on average. Minimising impacts and energy consumption throughout the product chain may be another essential element needed to reduce the environmental costs of fishing. Our results provided a set of recognised benchmarks that can be used for monitoring progress in this field.

## Abstract (summary)

**Table:** 

Measurement(s)	vessel speed • latitude • longitude • fuel consumption • shaft rotational speed • shaft torque • fishing gear drag
Technology Type(s)	GPS navigation system • mass and volumetric flow meter • shaft power measurement system with optical sensor and strain gauge
Factor Type(s)	fuel usage • greenhouse gas emissions
Sample Characteristic - Environment	greenhouse gas • fossil fuel
Sample Characteristic - Location	Mediterranean Sea

## Background & Summary

Globally, human activities vastly influence the earth’s climate and temperature^1,2^. Of major concern in this respect is the reduction of forests, livestock farming, and the burning of fossil fuels. To limit the impact of climate change and adhere to the goals of the Paris Agreement^3^, namely to limit the increase in global average temperature to well below 2 °C above pre-industrial levels, a swift and considerable reduction of emissions is indispensable.

Marine active fishing gear fisheries are energy-intensive food production methods, and their economic sustainability is very sensitive to fuel use^4^. Advances in fishing technology have also caused the motorisation of fishing fleets with more powerful engines and the increased demand by fisheries for fossil fuels^5,6^. This requires the maximisation of energy efficiency as fuel consumption by fishing vessels is typically the dominant driver of energy demand and greenhouse gas (GHG) emissions from fisheries production, accounting regardless of the gear used or species targeted for between 60 and 90% of emissions up to the point of landing^4,7^. While the inadequate techniques for analysis make it challenging to rank fishing gears and practices by their GHG emissions, relative fuel consumption across methods offers a reasonable surrogate for emissions^8^. Indeed, trawl fishing vessels, especially in the Mediterranean, tend to be exceptionally energy-inefficient, and approaches to enhance their energy efficiency would benefit the competitiveness and profitability of the fishing industry and the environment conservation^9–11^. The combustion of fossil fuels for human activities produces emissions of various GHG, including carbon dioxide (CO2), carbon monoxide (CO), oxides of nitrogen (NOx), sulphur dioxide (SO2), and non-methane volatile organic compounds^12^. A primary goal of the Paris agreement is to achieve sustainable management of natural resources to reduce GHG emissions and, in particular, reduce the emissions of CO2 from fossil fuel combustion. Trawling is an energy-intensive activity, and its economic sustainability is very sensitive to fuel consumption. At the same time, energy-efficient technologies and behavioural change can also decrease the damage to aquatic ecosystems, reduce emissions and lower fuel costs of capture fisheries^13–23^. The reduction of GHG emissions and the efficient use of resources have become critical political objectives on the agenda of the European Union^9,24^. Good energy performance of the fleets is essential to achieve economically and environmentally sustainable fisheries^4^.

Energy audits are effective ways to obtain a clearer idea of how energy is used in a business and subsequently identify ways of reducing energy consumption levels and associated costs^4,25^. Therefore, the adoption of an energy audit should be seen as one of the strategies that can be used to improve the outcomes for a fishery operating within an Ecosystem Approach to Fisheries (EAF) based management system^26^. For this reason, in the current study, an energy audit process for fishing vessels was developed and then trialled on several different fishing vessels. The EAF concept is a promising approach toward integrated environmental and fishery regulation^27–29^, but the energy implications have been neglected^6,30^. This is particularly problematic because fuel consumption is also linked to seafloor impacts. As stated by Thrane^31^, addressing fuel consumption may simultaneously address several other environmental problems in modern fisheries. Improvements in energy efficiency can reduce the need for investment in energy infrastructures, cut fuel costs, increase competitiveness, and decrease the negative environmental impact of fishing^4^. This shows that administrations have essential tools to pursue sustainable and energy efficient fisheries by directly influencing the energy costs or indirectly introducing carbon quotas, such as the European Union Emissions Trading Scheme^32^. Energy efficiency audits can serve as a tool for assessing the performance of the fleets, as well as the success of the innovative techniques applied^25^. As the future remains quite uncertain and expectations of further oil and fuel price increases are probable^4,25^, actions need to be taken to prepare for future fuel price increases and ensure economically, environmentally and socially sustainable use of fisheries resources.

Introducing Energy Audits to fishing vessels constitutes a practical approach to counteract energy inefficiency^5,6,10^. A vessel energy audit assesses how much energy is consumed by individual components of the vessel, including the propulsion system, AC and DC electrical and hydraulic circuits, as well as cooling equipment.

An energy audit allows for:

- establishing an energy consumption baseline;
- estimating the energy consumption of each component;
- allocating energy consumption in relation to specific vessel activity (e.g., sailing, searching for fish, or towing).

This analysis allows for identifying weaknesses in a targeted way enabling the identification of tailored solutions and remedies. Herein, opportunities arise through the availability of new technologies and products that reduce fuel consumption^33^ and lower exhaust emissions. Even simple measures can be effective, for example, other experiments^10,11^ showed a fuel savings of up to 15% obtained by reducing the steaming speed by half a knot. A reduction in fuel consumption by 15% represents millions of litres of fuel saved globally, which in turn translates into a considerable reduction in emissions and increased profitability for the fishing industry.

In an energy audit, sensitive instrumentation records fuel flow, shaft speeds, torque, AC and DC current flow, radiated heat, hydraulic fluid flow, and other parameters. The acquired data is analysed to identify wasteful high-energy-consumption components, which underpin energy conservation measures.

Current interest in developing energy efficiency strategies for the fishing industry, including alternative fuels and lubricants, has been triggered by a renewed rise in fuel prices and a concern for climate change. Attaining energy efficiency requires a carefully designed, comprehensive and coherent analytical approach^34^, a condition that energy audits can fulfil. The cornerstone of energy audits for fishing vessels lies in the continuous monitoring of their energy performance. As a result, wasteful energy consuming components can be identified, and energy efficiency-enhancing measures can be proposed^5^. Moreover, as part of a business plan, the energy profile of the vessel can be evaluated to understand how profitability levels can be increased by taking energy efficiency-enhancing measures. Energy audits help provide sustainability both on an environmental and an economic level. As in the proverbial “*if it pays, it stays*”, a solution that reduces fuel consumption, net of initial green investments to pay off, will also reduce running costs, which constitutes an incentive for its adoption.

Here, we draw upon this emerging topic to provide an overview of the current state of research on energy use in trawl fisheries. This paper describes the Mediterranean trawl fleets and addresses some questions dealing with its management. Even though the primary focus is on the Mediterranean, some considerations on environmental issues concerning energy use can be broadly scaled-up to other regions in the world with similar fleet structures. Coupled with concern over GHG emissions from fossil fuel combustion, greater focus is now being placed on energy-intense fisheries. Therefore, applying an energy audit may be the first important step toward systematically evaluating fuel-saving practices’ potential cost and environmental impacts on all fisheries. The Mediterranean context is fairly typical of the small-scale fishing industry in the European region. Labour costs are generally low, and fuel consumption may comprise a full 37% of the expenses for trawl fishing activities^10,35^. Therefore, reducing fuel use provides multiple economic and environmental benefits, and these positive results could be helpful to other countries.

Herein, we present the results of an analytical synthesis of data and energy performance indicators to identify fuel use patterns in fisheries targeting different species and employing different gears. A standard energy audit tool was conceived based on former experience with energy monitoring systems onboard fishing vessels^10,11^. To test value and efficiency, several energy audits were carried out between June 2008 and July 2018 on-board midwater pair trawlers (PTM), single boat bottom otter trawlers (OTB), and Rapido beam trawler (TBB), three major trawl fleet segments of the Mediterranean^36,37^. The primary goals of this work are, therefore:

- to apply, on a test fleet, the energy audit approach for fishing vessels, assessing its feasibility, effectiveness and value;
- to gather baseline data for energy cost analyses;
- to provide fishing vessel owners information on their vessel’s fuel energy use baseline along with the energy consumption of each vessel component and activity; and
- to help the owners identify feasible and cost-effective energy conservation measures.

## Methods

### Vessels monitored and on-site investigations

The current study has been conducted mainly to investigate energy use to subsequently identify potential ways to reduce energy consumption. Intuitively, as the pool of energy audit information on Mediterranean fishing vessels grows, it should be possible to determine which areas of research and development are most needed and embark on a long-term program to build up the necessary pool of technical expertise.

Ten vessels were monitored for tests, representing three main fleet sectors of the Mediterranean fisheries. We monitored two single boat bottom otter trawlers (OTB), seven midwater pair trawlers (PTM), and one Rapido beam trawler (TBB). Table 1 shows the main technical characteristics of these fishing vessels. Following the selection of the vessels, an energy audit template was developed to assess the main features of the vessels during fishing trips (e.g., engine, propeller and gear characteristics, hull type and design).

**Table 1:** Main characteristics of the monitored fishing vessels.

Vessel	Audit or monitoring dates Year(months)	VL	LOA [m]	LPP [m]	B [m]	D [m]	GRT [GT]	PB [kW]	RPM [rpm]	R [−]
**OTB01**	2011(1,6)	VL1824	21.5	17.0	5.7	1.8	82	478	1,600	5.6
	2015(7,8)									
**OTB02**	2011(2,7)	VL1824	22.8	19.6	6.2	1.8	91	574	1,600	5.0
**TBB01**	2016(1–12)	VL2440	25.9	20.6	6.6	2.2	86	884	1,600	5.9
	2017(1–7, 9–12)									
	2018(1–7)									
**PTM01**	2011(2)	VL2440	28.6	21.2	6.9	2.2	99	940	1,800	6.3
**PTM02**	2011(4)	VL2440	29.0	24.3	6.9	2.2	138	940	1,800	5.0
**PTM03**	2011(7)	VL2440	26.5	20.9	6.8	2.2	96	870	1,600	5.9
**PTM04**	2011(10)	VL2440	25.5	20.1	6.6	2.0	132	772	1,800	5.5
**PTM05**	2012(7)	VL2440	25.9	20.6	6.6	2.2	86	884	1,600	5.9
**PTM06**	2008(6, 7, 9–11)	VL2440	29.0	24.3	6.9	2.2	138	940	1,800	5.0
**PTM07**	2008(5–7, 9–11)	VL2440	27.0	20.6	7.0	2.0	139	809	1,800	5.5

Dates (years and months, in parenthesis) of the audits and on-site investigations are reported for each vessel (OTB: single boat bottom otter trawler; PTM: midwater pair trawler; TBB: Rapido beam trawler). LOA: vessel length overall; LPP: length between perpendiculars; B: beam; D: propeller diameter; GRT: gross register tonnage; PB: installed engine brake power; RPM: maximum propeller shaft revolution per minute; R: gearbox ship reduction ratio. Vessel length segment (VL) is assigned based on LOA (VL0612: vessel between 6 and 12 m; VL1218: vessel between 12 and 18 m; VL1824: vessel between 18 and 24 m; VL2440: vessel between 24 and 40 m).

The duration of a fishing trip or monitoring is affected by different variables, such as target species, fishing gear, and weather conditions. The fishing trips are relatively constant by type of fishery throughout weeks of the year. In an ordinary week, both OTB and TBB vessels leave port on Monday morning and return on Thursday morning. The duration of PTM vessels is also considerably constant. They usually have daily trips from Monday to Thursday, with vessels leaving the harbours early morning and returning late afternoon. For all fisheries, the active fishing days are from Monday to Thursday as from Friday to Sunday fishing is not allowed (Table 2) in Adriatic.

**Table 2:** Type of activity in a 24-hour day during an ordinary working week.

Hour/Day	OTB, TBB				PTM	
	Mon	Tue-Wed	Thu	Week	Mon-Thu	Week
1	H	T	T		H	
2	H	T	T		H	
3	S	S	T		H	
4	S	H	T		H	
5	T	S	T		S	
6	T	S	T		S	
7	T	T	T		T	
8	T	T	S		S	
9	T	T	S		S	
10	T	T	H		T	
11	T	T	H		S	
12	T	T	H		S	
13	S	S	H		T	
14	T	T	H		S	
15	T	T	H		T	
16	T	T	H		S	
17	T	T	H		H	
18	T	T	H		H	
19	T	T	H		H	
20	T	T	H		H	
21	T	T	H		H	
22	T	T	H		H	
23	T	T	H		H	
24	T	T	H		H	
**Harbour (H)**	**2**	**1**	**15**	**19**	**12**	**48**
**Sailing (S)**	**3**	**4**	**2**	**13**	**8**	**32**
**Towing (T)**	**19**	**19**	**7**	**64**	**4**	**16**

Hours of activities (in harbour, H; steaming, S; towing, T) are specified for each vessel type (OTB: single boat bottom otter trawler; PTM: midwater pair trawler; TBB: Rapido beam trawler). For all fisheries, the active fishing days are from Monday to Thursday as from Friday to Sunday fishing is not allowed in Adriatic.

### Energy audit framework

The energy audit was carried out in four steps:

1. preliminary interview with fishers. This was necessary to collect information about vessel characteristics such as size, power, propulsion system characteristics, target species, crew, machinery etc.;
2. installation of the measurement kit on the vessel;
3. monitoring of energy-consuming components and data recording with customised software during fishing trips;
4. post-processing and data analysis to calculate energy performance indicators during steaming and towing to establish the energy profile of the vessel.

**Fig. 1:**
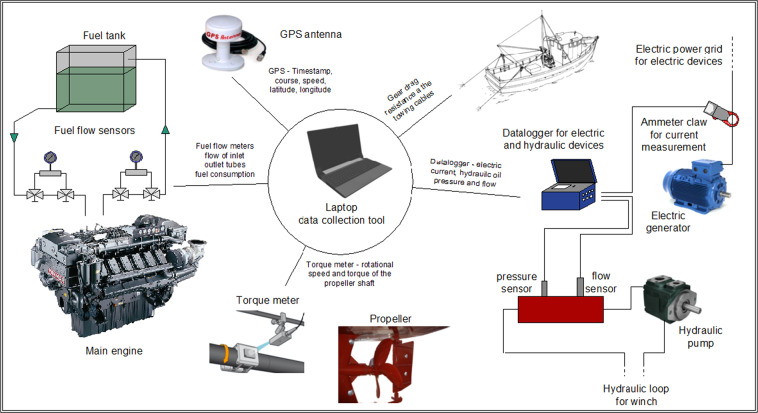
Measurement kit layout for energy audit in fisheries. Data collection system used for the on-site vessel investigations for a detailed analysis of energy consumption during typical commercial fishing trips. The system consists of two flow meters for fuel consumption, a shaft power meter, a hydraulic and electric power analyser, two load cells for towing drag resistance, and a GPS data logger. Serial communication ports RS232/485 link the instruments to a laptop, automatically controlling data acquisition.

On-site vessel investigations for a detailed analysis of energy consumption were conducted during typical commercial round trips, which for trawlers consist of various activities (e.g., sailing, searching for fish, or towing). The data collection system, conceived at the National Research Council (CNR), consists of two flow meters for fuel consumption, a shaft power meter, a hydraulic and electric power analyser, two load cells for towing drag resistance, and a GPS data logger. Serial communication ports RS232/485 link the instruments to a computer, which automatically controls data acquisition. Figure 1 shows the measurement kit layout.

### Engine fuel usage

**Fig. 2:**
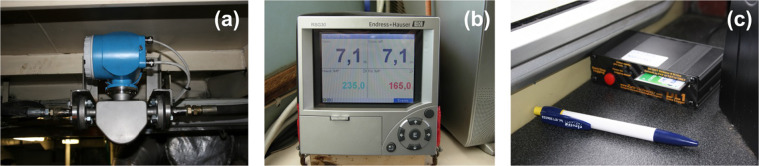
Engine fuel efficiency system mounted onboard the monitored fishing vessels. (**a**) mass flow sensors for fuel consumption measurement; (**b**) multi-channel recorder mounted on the vessel’s bridge to visualise the fuel consumption; (**c**) GPS data logger.

At the beginning of the experiment, we investigated the accuracy, precision, and robustness of different fuel flow meters, establishing the most accurate way of measuring fuel consumption and how the devices should be fit. We also tested whether the sensors were coping with the general conditions on fishing vessels. The main metering device selected consisted of two Coriolis mass flow sensors, one multichannel recorder and one GPS data logger (Fig. 2a). Both flow sensors were connected to a multichannel recorder (Fig. 2b), which showed the fuel consumption rate [*l/h*] as well as the total fuel consumption [*l*].

The Coriolis measurement does not depend on the fluid’s physical properties, such as viscosity and density. To accurately measure both the instant and total fuel consumption, the mass flow sensors were positioned at the inlet and outlet of the main vessel engine. This setting ensured that sensors measured the fuel used by the propulsion system and other power demanding components, e.g. pumps, generators etc., which are usually connected to the main engine. The Coriolis meter, the type of sensor used for this study, is a sensible choice when fuel consumption rates are above 25 l/h, especially if there is a substantial return flow to the tank from the engine. As Coriolis meters measure the mass flow rate, there is no need to apply a temperature correction as for common turbine meters. Even if the temperature increase in the outlet fuel line is significant, Coriolis meters provide precise and accurate fuel consumption measures^10^.

Following the technical specifications on the flow meter datasheet, the maximum measured errors of reading (*mme*) for different operating conditions can be calculated:

$$mme=\pm 0.70 \% \pm \left[\left(zps/mv\right)\times 100\right] \% $$

where *zps* is the zero-point stability, and *mv* is the measured value. Concerning the installed Coriolis sensors, which have zero-point stability of 0.20 l/h, the maximum measured errors yield 2.7% of readings for the minimum flow of 10 l/h. However, under normal trawling and sailing conditions, where the mean flows are ≥50 l/h, *mme* are ≤1.1% of readings.

**Fig. 3:**
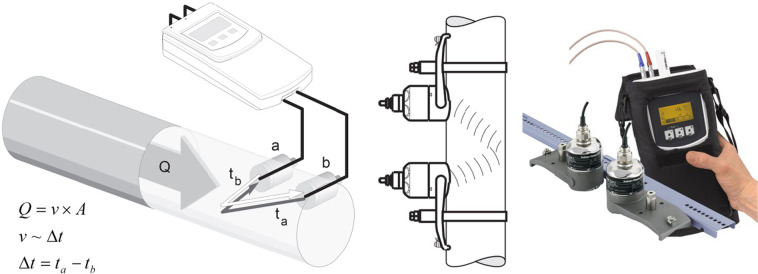
Measuring principle and mounting arrangement of the portable ultrasonic flow meter. The system has two acoustic sensors (**a**,**b**) for measuring the volume flow (*Q*) of the fluid from the cross-sectional pipe area (*A*) and the flow velocity (*v*) obtained by the transit time difference (*Δt*).

Besides fuel consumption, geo-referenced positions, and speed of each haul were simultaneously collected. The GPS logger unit recording latitude, longitude and speed does not include an in-vehicle display (Fig. 2c). It comprises a data logger and an 8-channel GPS receiver connected with an external antenna. Data were stored at a rate of 1 second on compact flash memory devices and were periodically downloaded for the data elaboration. For two vessels (PTM03 and OTB02), the effective fuel consumption was measured by two portable ultrasonic flow meters (Fig. 3). The measuring system consists of one transmitter and two sensors. In this measurement method, acoustic (ultrasonic) signals are transmitted between the two sensors. The system is based on the principle of transit time difference. The signals are sent in both directions, i.e. the sensor works as both a sound transmitter and a sound receiver (Fig. 3). As the propagation velocity of the waves is less when the waves travel against the direction of flow than along the direction of flow, a transit time difference occurs. This transit time difference is directly proportional to the flow velocity. The measuring system calculates the volume flow of the fluid from the measured transit time difference and the cross-sectional pipe area. In addition to measuring the transit time difference, the system simultaneously measures the sound velocity of the fluid. This additional measured variable can be used to distinguish different fluids or to determine fuel quality.

The measured error for these ultrasonic flow meters depends on several factors. A distinction is made between the measured errors of the device, which is 0.5% of the measured values) and an additional installation-specific measured error (typically 1.5% of the measured value) independent of the device. The measured installation-specific error depends on on-site installation conditions, such as the nominal diameter, wall thickness, pipe geometry, fluid etc. The sum of the two measured errors is the maximum measured error at the measuring point. Given a flow velocity of >0.3 m/s and a Reynolds number >10000, the typical error limits: ± 2% of reading ± 0.05% of full scale, which corresponds to a value of 10 m/s for the installed ultrasonic devices.

### Propulsion system

**Fig. 4:**
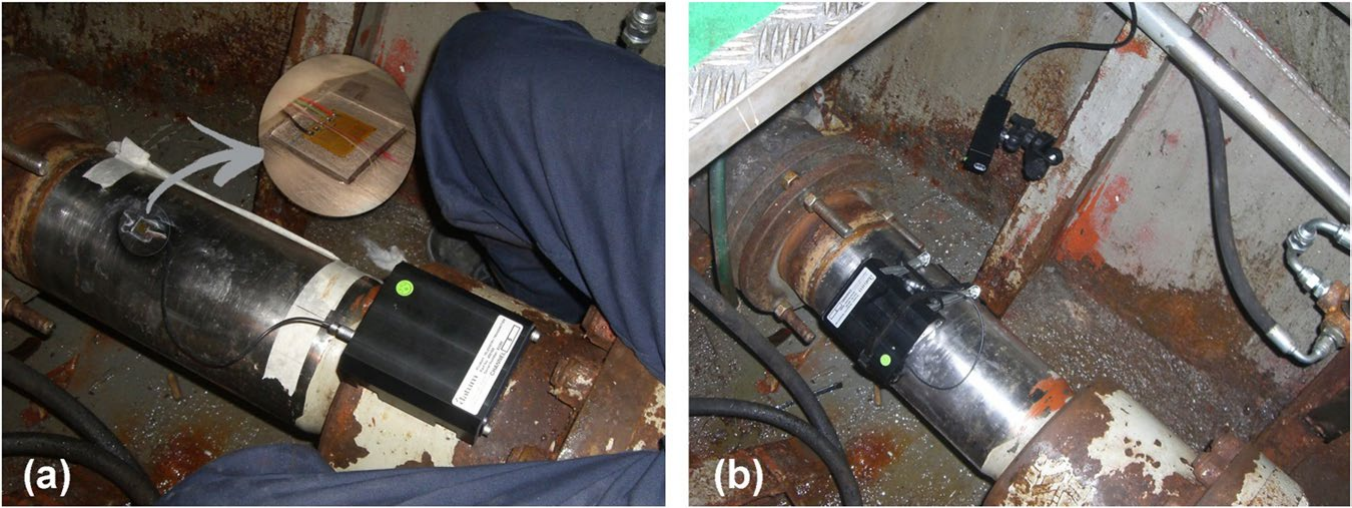
Torque meter and video camera RPM counting device. Both apparatuses are used for the shaft power evaluation: (**a**) magnifier glass showing the strain gauge installed on the propeller shaft and connected to the data acquisition box; (**b**) video camera used to transmit the torque and rotational speed to a personal computer by an RS232/485 serial port.

The power delivered by the main engine to the propeller for the propulsive thrust is measured with a shaft power meter equipped with a battery-powered shaft-mounted strain gauge (Fig. 4). The propeller-shaft torque transducer measures the surface tension at the shaft through a strain gauge, configured as “Wheatstone bridge” and utilises a short-range radio transmission for the data transfer to the receiver off the shaft. The propeller-shaft torque transducer utilises a short-range radio transmission for the data transfer from the rotating shaft to the receiver off the shaft. The recorder measures shaft rotational speed through an optical proximity sensor. The system opens the opportunity to collect data accurately in the field, without the need to disrupt and modify the shaft. The strain gauges used are supplied with the connector to remove the need for soldering and have an encapsulated coating to simplify environmental sealing. According to the technical documentation, the instrumentation has a reading accuracy of 0.1%.

### AC electrical and hydraulic systems

**Fig. 5:**
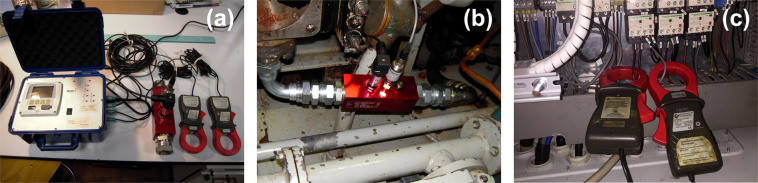
AC electric and hydraulic data collection system. (**a**) Complete system; (**b**) hydraulic sensor measuring the flow and pressure from the hydraulic pipeline; and (**c**) clamp-on ammeters measuring the electric power supply from the alternator.

Electric and hydraulic power data acquisition is performed by a single data logger (Fig. 5a). The hydraulic power analyser consists of a sensor array that provides flow and pressure from the main hydraulic pipeline (Fig. 5b). The electric power supply from the alternator is measured by two clamp-on ammeters (Fig. 5c). The instrument provides a one-point calibration that can eliminate the instrument’s accuracy failures. The technical specification datasheets declare the accuracy of <1% for pressure and electrical measurements.

### Towing drag efficiency

**Fig. 6:**
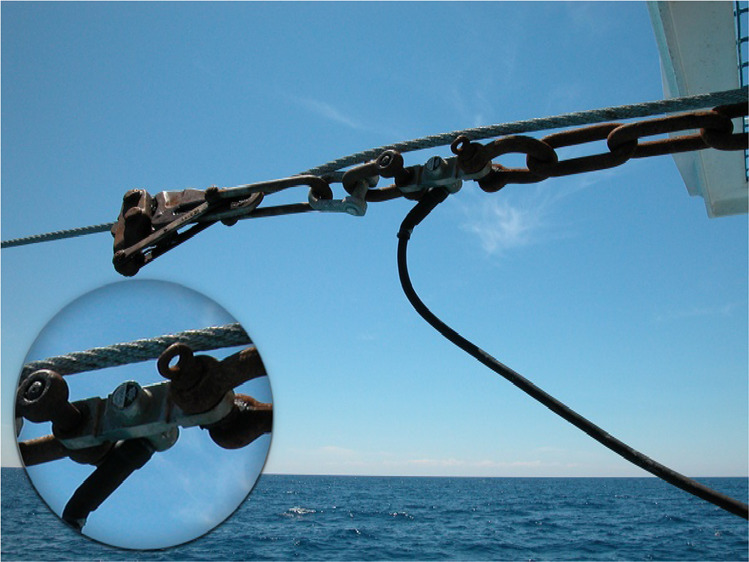
Load cell for total gear drag measurement. Two electronic load cells have been used to measure the warp loads during towing conditions.

Two electronic load cells measure the warp loads during towing activities. According to the technical specifications, the measuring cells mount a temperature compensated strain gauge with a resolution of 2.2 kg and an accuracy of 25 kg. After shooting the gear, load cells are mounted on the towing warps to measure the total drag resistance of the fishing gear (Fig. 6) at a measuring rate of 1 s.

### Software and code availability

The tested data collection system, conceived at the CNR, consists of a portable laptop, which automatically controls data acquisition and provides correct real-time functioning of the vessel monitoring through customised software. The data processing software is written in Microsoft Visual Basic, and data storage and management are ensured through a Microsoft Access database. Code and database structure are available upon request, and complete documentation and advice on extending the application to other fisheries.

### Energy and GHG emission performance

The total energy consumption results from a complex set of interacting components and actions during fishing trips. These are relevant in terms of costs and benefits and business profitability, contributing to a comprehensive picture of the energy input and output.

A new and customised indicator, named energy performance indicator (*EPI)*, is introduced to compare fishing methods where the same species is targeted, possibly in the same region. More efficient vessels have higher *EPI* values, which are calculated as the ratio between the propulsion power delivered to the propeller, *PS[kW]*, and the thermal power of the burned fuel, *PF[kW]:*

1 $$EPI=PS/PF\times 100$$

with

2 $$PS=RPS\times kM$$

and

3 $$PF=fc\times \rho \times LHV$$

where *RPS* in Eq. (2) is the intermediate propeller shaft revolutions per second and computed as:

4 $$RPS[rad/s]=RPM[{{\rm{\min }}}^{-{\rm{1}}}]\times 6.28/60$$

While *kM* in Eq. (2) is the intermediate propeller shaft torque in [kNm] units:

5 $$kM[kNm]=M[Nm]/1000$$

The fuel consumption, *fc[l/s]* in Eq. (3), originates from the measured fuel consumption of the main engine, *hFC[l/h]*, and is computed as:

6 $$fc[l/s]=hFC[l/h]/3600$$

According to the standards ISO 3675:1998^38^, the diesel density varies between 0.820 and 0.890 kg/l, in Eq. (3) we assumed for our computation a mean value of $\rho \left[kg/l\right]=0.860$.

*LHV* in Eq. (3) is the *Lower Heating Value* of the diesel, which according to the ISO 8217:2017^39^ is 42.7 [kJ/kg]:

7 $${LHV}[kJ/kg]=42.7\times 1{0}^{3}$$

The lower heating value (also known as net calorific value) of a fuel is defined as the amount of heat released by combusting a specified quantity (initially at 25 °C) and returning the temperature of the combustion products to 150 °C, which assumes the latent heat of vaporisation of water in the reaction products is not recovered^40^. Noteworthy, *EPI* only accounts for the energy consumption of the main propulsion system as in other studies^11,41^ have demonstrated that neither the electric nor the hydraulic components considerably influence the total consumption balance of Mediterranean trawlers^11,41^.

Concerning the GHG emissions associated with fuel combustion, it is essential to know that they are a function of: i) the volume of fuel combusted, ii) the density of the fuel, iii) the carbon content of the fuel, and iv) the fraction of carbon that is oxidised to CO2^42–44^. Petroleum diesel is produced from the fractional distillation of crude oil at 200–350 °C, resulting in a mixture of carbon chains that typically contain between 9 and 25 carbon atoms per molecule^45^. For our computations, we assumed 15 carbon atoms per diesel molecule. As the polycyclic aromatic hydrocarbons have the chemical formula *C*_*n*_*H*_*2n*_^46^, the molar mass of a molecule *C*_15_*H*_30_ is, therefore:

8 $${C}_{15}{H}_{30}[g/mol]=12\times 15+1\times 30=210$$

where 12 and 1 in the formulae of Eq. (8) are the standard atomic weights of the carbon and hydrogen, respectively^46^. Considering a mean density of 860 g/l, 1 litre of diesel corresponds to 4 mol of *C*_15_*H*_30_ (i.e., 860/210≃4), or else to 60 mol of carbon (i.e., 4 × 15 = 60), where 15 are the number of carbon atoms per diesel molecule.

A simplified equation for the combustion of a hydrocarbon fuel may be expressed as follows:

9 $$Fue{l}_{\left({C}_{n}{H}_{2n}\right)}+Oxige{n}_{\left({O}_{2}\right)}\to Wate{r}_{\left({H}_{2}O\right)}+Carbon\;Dioxid{e}_{\left(C{O}_{2}\right)}+{Heat}$$

In the combustion reaction of Eq. (9), the process produces heat that is converted into mechanical energy, while the hydrogen from the fuel combines with oxygen from the air to produce water (*H*_2_*O*) and carbon dioxide (*CO*_2_). Hence, burning 1 litre of diesel (i.e., 60 mol of carbon) produces an equivalent quantity of 60 mol of carbon dioxide, which have an overall weight of:

10 $$C{O}_{2}[g/l]=60\times \left(12+16\times 2\right)=2640$$

where 16 is the atomic weight of the oxygen. Based on the information available on the fuel being consumed *hFC*[l/h], the appropriate equation to calculate the fuel-related GHG emissions (e.g., CO2-eq per litre of fuel based on the chemical content of marine fuels) in an hour is as follows:

11 $$hGHG[kg/h]=hFC[l/h]\times 2640\left[g/l\right]\times 1{0}^{-3}$$

This indicator is a linear function of energy use and, therefore, performs similarly. Thus, in the current study, fuel use and carbon footprint comprise the emissions from capture to landing and do not account for post-landing emissions, including processing, packaging and transportation inputs.

### Data analysis

For each fishing activity (e.g., sailing or searching for fish and fishing), the data analysis has included the identification of homogeneous load conditions of the engine (namely field *Dval* in the dataset, see Table 3), for which we calculated mean values of the main parameters (e.g., *SOG*, *RPM, M*, *PS*, *PF*, *FT*, *hFC*, and *hGHG*). All these parameters and the *EPI* indicator were also modelled against mean speed to estimate standardised average values: 1) at a fixed speed of 10 kn under steaming conditions and; 2) at vessel-specific resulting mean speed during towing. Since fuel consumption is the most relevant parameter, the mean values (litres/hour) at steaming and towing conditions were correlated and plotted against mean vessel speeds.

**Table 3:** Data field definitions.

Field	Unit	Description
Code	(—)	Vessel code, see Table 1 for main characteristics
Date	dd/mm/yyyy	Date of the audit or monitoring work, see Table 1
Time	(hh:mm:ss)	Acquisition time. In post-processing, the raw data have been time-averaged at 10 s interval
IDActivity	(—)	Main vessel activity. 1: sailing or searching for fish (steaming); 2: towing
Haul	(—)	Progressive number of the haul *(only during towing)*
DVal	(—)	Progressive number identifying homogeneous load conditions of the main engine and vessel activities
SOG	[kn]	Vessel speed over ground
COG	[°]	Vessel course over ground *(in 360 degrees)*
Lat	[dd.mm]	Latitude in decimal degrees *(six-decimal degrees)*
Long	[dd.mm]	Longitude in decimal degrees *(six-decimal degrees)*
hFC	[l/h]	Measured fuel consumption of the main engine
FT	[kg]	Towing gear drag *(only during towing)*
M	[Nm]	Intermediate propeller shaft torque
RPM	[rpm]	Intermediate propeller shaft revolutions per minute
PS	[kW]	Propulsion power *(measured at the intermediate shaft)*
**Metric**	**Unit**	**Description**
PF	[kW]	Thermal power of the burned fuel
dGHG	[kg CO2/day]	Greenhouse gas emission rates, equivalent CO2 emission (CO2-eq) in an ordinary fishing day, week, or year, respectively
wGHG	[kg CO2/week]	
yGHG	[kg CO2/year]	
dFC	[l/day]	Calculated fuel consumption rates in an ordinary fishing day, week, or year, respectively
wFC	[l/week]	
yFC	[l/year]	
EPI	(%)	Energy performance indicator, ratio between propulsion power (PS) and thermal power of the burned fuel (PF)
FUI	[l/t]	Fuel use intensity (litres of fuel per ton of landed fish)
CF	[kg CO2/t fish]	Carbon footprint (kg of CO2-eq per ton of landed fish)

Codes of the parameters used in the Energy audit data collection and post-processing (Field), and definition of the main energy metrics (Metric) estimated in the analysis. The Energy audit dataset is available through the unrestricted repository at *Figshare*, see Sala *et al*.^54^.

For each vessel, annual catch data and fuel consumption have been then used to calculate fuel use intensity (FUI) as typically expressed in terms of litres of fuel burned per ton of live weight landings^47^ and carbon footprint (CF) in terms of kg of CO2-eq/ton of fish landed^47^. Fuel consumption can generally be used as a proxy for fishery carbon footprints, allowing for reasonable estimates without the time and effort required for a full life cycle assessment (LCA) study^47–49^.

### High-resolution logbooks and landing declarations dataset

To increase the level of detail, a complementary high-resolution logbook dataset of direct observations, collected in 2019 by scientific personnel on 45 commercial fishing vessels (19 OTB, 8 TBB, and 18 PTM), containing landings and fuel consumption information, was combined with the on-site energy audits. The Electronic logbook is the key element of the Electronic Recording and reporting System (ERS) defined within the European Fisheries Control Framework^50–52^ used to record, report, process, store and send fishery data (catches, landings, sales and transhipment). The analysed logbook 2019 data were thus effort (in active fishing days), fuel consumption, and annual landings overall and by species, which allowed the computation of FUI and CF of each fishing vessel. To obtain fisheries-specific fuel use estimation, the combined dataset (e.g., energy audits and high-resolution logbook dataset) was used to model the relationship between daily fuel consumption and vessel length overall (LOA). This theoretical LOA-based fuel use model, responding to the combined analysed dataset, was then scaled up to infer the daily fuel consumption of the entire national fleet/segments.

### Cross-analysis of fuel data with the scientific Fisheries Dependent Information (FDI) dataset

As abovementioned, the theoretical LOA-based fuel use model was applied to the Scientific Fisheries Dependent Information (FDI) effort dataset to infer specific fuel consumption per fishing day (including steaming and towing) for each fishery and vessel segment. National FDI landings were matched to the effort data, hence fuel consumption, to allow the computation of FUI and CF at the entire fleet and vessel segments level.

Annual fishing fleet effort and landing 2019 data of the entire national trawls fleet were obtained from the FDI database, made freely available in aggregated form for ease of access by the Joint Research Centre (JRC) data dissemination tool, with detailed landings by gear, species and area of capture. The FDI database is updated annually and published at https://stecf.jrc.ec.europa.eu/dd/fdi together with information on the data-handling procedures. The JRC data dissemination tool provides access to data submitted by the EU Member States to the European Commission under the provisions of the Data Collection Framework (DCF)^53^. Fishery data are collected by the EU Member States based on national sampling programmes, implementing the EU Common Fisheries Policy (CFP).

## Data Records

For each monitored vessel trip of this study, raw data were stored at a rate of 1 s on hard disks and downloaded at the end of each audit or vessel monitoring for data elaboration. First, a data cleansing process was performed interactively with data wrangling tools or as batch processing through scripting to detect and correct corrupt or inaccurate records. The inconsistencies detected may have been initially caused by corruption in transmission or measurement instruments. Inaccuracy of a single measurement may have been considered acceptable, and related to the inherent technical error of the measurement instrument. Hence, data cleansing focused only on errors beyond minor technical variations, which constitute a significant shift within or beyond the population distribution.

After cleansing, raw data have been time-averaged at 10 s intervals to hold them in a Microsoft Access database. Routines have been finally specifically written to export the time-averaged data into an elaborated ASCII file and made available through an unrestricted repository at *Figshare*^54^ as a Comma-Separated Values (CSV) file. The dataset comprises 15 fields that collectively describe the sailing patterns or searching for fish and towing activities associated with the energy consumption and fuel-related GHG emission. All field codes and definitions are described in Table 3 to facilitate data re-use and re-processing. Additionally, the elaborated Microsoft Excel file of the high-resolution logbooks^55^ and the FDI files containing fishing capacity, effort, and catch data^56^ have also been made available through unrestricted repositories at *Figshare*.

## Technical Validation

### Energy audits

The present energy audits dataset, including unpublished earlier versions, provides a valuable resource for further research. Energy audits enable companies to know their status concerning energy use. In fisheries, they provide a detailed scan of the energy flows of each specific activity and propose measures to help reduce the energy demand, hence resulting in economic and environmental savings^57^. The established baselines on energy usage and emissions present the findings in the form of measures against defined benchmarks. This benchmark data can be used for analysing performance across a fishery or between fisheries, both at a national and international level. Furthermore, such data will benefit a range of parties interested in energy-efficient fishing, namely fisheries managers, government organisations, and bodies of conservation interest.

Other energy audit studies or publications that address the utilisation of fuel energy by the fishing industry^4–7,47,48,57–62^ can provide helpful information on energy use and CO2 equivalent emissions in other fisheries and can be used to support the technical quality of the current datasets.

The activity patterns of fuel consumption, GHG emissions, thermal power of the burned fuel and the resultant power delivered are listed in Table 4, with their associated energy performance indicator (*EPI*). This information will prove insightful to a wide spectrum of people, ranging from proactive fishing vessel owners planning contingencies when diesel prices escalate and erode profits, to government, industry advisers and decision-makers committed to securing a future for an industry that is very reliant on fuel to harvest valuable fish resources. According to the results obtained in the present study, the Rapido beam trawler targeting common sole (*Solea solea*) and purple dye murex (*Bolinus brandaris*) is overall the least efficient (rank 10, Table 4) whilst, except for two vessels (PTM3 and PTM5), the midwater pair trawlers targeting small pelagics, such as European anchovy (*Engraulis encrasicolus*) and sardine (*Sardina pilchardus*), are the most efficient fishing vessels.

**Table 4:** Estimated values of the main parameters and energy metrics.

Vessel	SOG [kn]	hFC [l/h]	wFC [l/week]	wGHG [kg CO2/week]	FT [kg]	PS [kW]	PF [kW]	*EPI* (%)	Rank
**Sailing**									
PTM07	10	81.2	2,597	6,857	—	432	828	52.1	1
PTM06	10	91.2	2,918	7,703	—	461	930	49.6	2
PTM02	10	81.6	2,611	6,893	—	405	832	48.7	3
PTM01	10	99.0	3,169	8,365	—	330	690	47.8	4
OTB02	10	66.0	858	2,265	—	285	673	42.3	5
PTM04	10	78.8	2,521	6,654	—	263	629	41.7	6
PTM05	10	65.6	2,099	5,542	—	219	558	39.3	7
OTB01	10	54.3	706	1,865	—	190	554	34.3	8
TBB01	10	91.5	1,190	3,141	—	308	934	33.0	9
PTM03	10	93.8	3,002	7,924	—	301	957	31.5	10
**Towing**									
PTM02	4.5	125.4	2,007	5,298	6,203	674	1,280	52.7	1
PTM06	4.4	133.5	2,137	5,641	6,064	703	1,362	51.6	2
PTM01	4.3	117.3	1,877	4,956	5,877	391	818	47.8	3
PTM07	4.4	129.3	2,069	5,463	6,035	631	1,319	47.8	4
PTM04	4.2	94.4	1,511	3,988	5,679	315	754	41.7	5
OTB02	3.8	74.2	4,748	12,533	4,105	307	757	40.6	6
PTM05	4.2	126.0	2,016	5,322	6,261	420	1,071	39.2	7
PTM03	4.8	91.7	1,468	3,875	5,291	363	936	38.8	8
OTB01	3.7	57.3	3,665	9,674	3,870	217	584	37.1	9
TBB01	6.9	120.5	7,709	20,352	5,957	376	1,229	30.6	10
**Overall**									
PTM06	—	105.3	5,054	13,344	—	542	1,074	50.4	1
PTM02	—	96.2	4,618	12,191	—	495	981	50.4	2
PTM07	—	97.2	4,666	12,319	—	498	992	50.2	3
PTM01	—	105.1	5,046	13,321	—	350	733	47.8	4
PTM04	—	84.0	4,031	10,643	—	280	671	41.7	5
OTB02	—	72.8	5,606	14,799	—	303	743	40.9	6
PTM05	—	85.7	4,115	10,864	—	286	729	39.3	7
PTM03	—	93.1	4,469	11,799	—	322	950	33.9	9
OTB01	—	56.8	4,371	11,539	—	212	579	36.6	8
TBB01	—	115.6	8,899	23,493	—	365	1,179	30.9	10

The metrics are calculated at 10 kn of vessel speed during steaming (sailing or searching for fish) and at vessel-specific resulting mean speed during towing. The Overall values are weighted averages accounting the relative contribution, or weight, of the steaming and towing working hours (see Table 2). The ranking is based on the vessel energy performance indicator, *EPI*(%). Vessels are listed according to an ascending order of *EPI*, hence Rank. See Table 3 for specifications of the parameters and metrics. OTB: single boat bottom otter trawlers, PTM: midwater pair trawlers, TBB: Rapido beam trawlers (TBB).

**Fig. 7:**
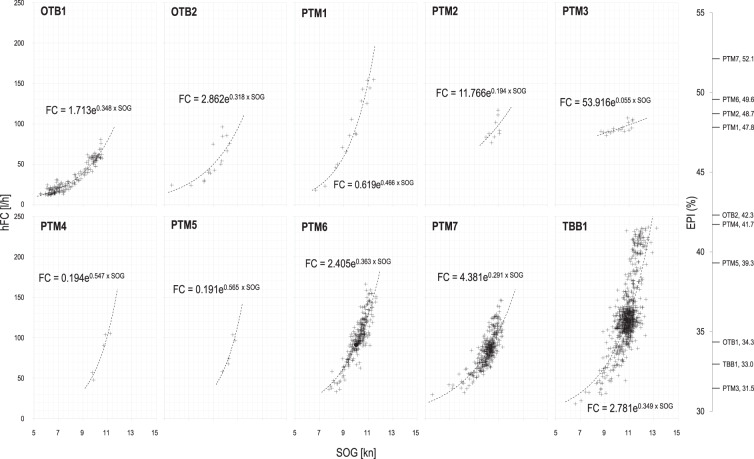
Mean fuel consumption, hFC[l/h], during steaming (sailing or searching for fish) conditions against vessel speed, SOG[kn]. Mean fuel consumption is calculated at each homogeneous load condition of the engine. The main characteristics of the monitored vessels (OTB: single boat bottom otter trawler; PTM: midwater pair trawler) are reported in Table 1. On the right-hand side, the standardised energy performance indicator *EPI* at 10 kn has been reported for each vessel. Higher is *EPI*, more efficient is the fishing vessel.

**Fig. 8:**
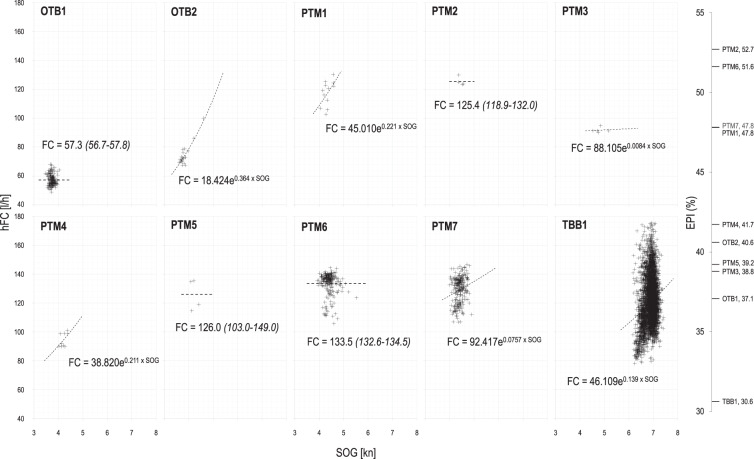
Mean fuel consumption, hFC[l/h], during towing activities against vessel speed, SOG[kn]. Mean fuel consumption is calculated at each homogeneous load condition of the engine. Main characteristics of the monitored vessels (OTB: single boat bottom otter trawler; PTM: midwater pair trawler) are reported in Table 1. On the right-hand side, the mean modelled value of the energy performance indicator *EPI* at vessel-specific resulting mean speed during towing has been reported for each vessel. Higher is *EPI*, more efficient is the fishing vessel.

Mean fuel consumption values plotted against vessel speed at homogeneous load conditions of the engine during steaming and towing activities are displayed in Fig. 7 and 8, respectively. All data recorded in a speed range typical for sailing or searching for fish (5–12 kn) were analysed for steaming conditions. The fishing vessels carried out several hauls during the monitored trips under different conditions, such as wind and waves strengths. To compare vessel performances, the mean modelled values of all parameters (*hFC, hGHG, PS*, *PF*, and *EPI*) at 10 kn for steaming, and at each vessel-specific mean speed for towing have been reported in Table 4. In general, midwater pair trawlers (PTM) and Rapido beam trawlers (TBB), both in steaming and towing conditions, tend to have higher power demand (*PS*) and thermal power (*PF*) of the burned fuel compared to OTB. However, except for PTM3, which resulted in worst performances with the lowest *EPI* in steaming (Table 4), their standardised *EPI* is higher, and therefore their efficiency.

### Fuel use intensity and carbon footprint per métier and model verification

Analysing catch and fuel consumption by fishing activity allows for more accurate estimates of fuel use intensity and carbon footprint induced by the various fleets. To make this approach operational, the first step is the definition of homogeneous groups of fishing vessels^63^. The establishment of the European Data Collection Framework (DCF)^64^ has adopted the definition that we follow here: a métier is a group of fishing operations targeting a specific assemblage of species, using a specific gear, during a particular period of the year and within the specific area. Therefore, the on-site energy audits and complementary high-resolution logbook datasets have been merged to define FUI and CF by métier.

Seven métiers have been identified as having similar gear, catch composition, fishing area, and resulting FUI and CF (Tables 5–7), and so in addition to its statistical scope, it also represents a major insight into the energy use intensity of Mediterranean trawl fisheries. Although time and space are implicitly part of the definition of a métier, the gear and target species represent the two main identifiers, with the variability due to time and space being more or less marked for the different gear types^65^. This is particularly evident for the bottom otter trawl targeting mixed demersal species, where we defined a single métier covering all the national waters (Table 5).

**Table 5:** Fuel use intensity (FUI) and carbon footprint (CF) in single boat bottom otter trawl (OTB).

Target species (Area)	Data source	Vessel ID	VL	LOA [m]	hFC [l/h]	dFC [l/day]	yFC [l fuel/year]	yGHG [kg CO2/year]	Landings [kg/year]	FUI [l fuel/t fish]	CF [kg CO2/t fish]
**Shrimp** (Strait of Sicily)	DCF	**OTB03**	VL2440	26.1	78.1	1,503	264,570	698,465	23,283	11,363	29,998
	DCF	**OTB04**	VL2440	26.8	88.5	1,704	299,978	791,941	27,985	10,719	28,299
	DCF	**OTB05**	VL2440	27.0	91.0	1,752	308,293	813,893	26,791	11,507	30,380
	DCF	**OTB06**	VL2440	29.0	102.6	1,975	347,658	917,818	30,778	11,296	29,821
	DCF	**OTB07**	VL2440	29.6	96.8	1,863	327,900	865,655	27,300	12,011	31,709
		**Mean** *(CI95%)*								**11,379** *(10,804*–*11,955)*	**30,041** *(28,523*–*31,560)*
**Mixed demersal** (All Italian seas)	DCF	**OTB08**	VL1824	18.1	42.3	815	143,382	378,528	31,797	4,509	11,905
	DCF	**OTB09**	VL1824	18.4	52.4	1,009	177,553	468,741	56,951	3,118	8,231
	DCF	**OTB10**	VL1824	19.3	58.4	1,125	197,998	522,714	50,511	3,920	10,349
	DCF	**OTB11**	VL1824	19.5	54.3	1,045	183,888	485,465	42,432	4,334	11,441
	DCF	**OTB12**	VL1824	20.4	49.4	950	167,237	441,507	31,682	5,279	13,936
	DCF	**OTB13**	VL1824	20.6	60.6	1,167	205,333	542,080	51,690	3,972	10,487
	DCF	**OTB14**	VL1824	20.9	55.3	1,064	187,257	494,359	36,638	5,111	13,493
	AUDIT	**OTB01**	VL1824	21.5	56.8	1,093	192,319	507,721	58,128	3,309	8,735
	AUDIT	**OTB02**	VL1824	22.8	72.8	1,401	246,644	651,139	65,803	3,748	9,895
	DCF	**OTB15**	VL2440	24.1	69.4	1,337	235,278	621,133	78,807	2,986	7,882
	DCF	**OTB16**	VL2440	24.5	79.7	1,534	269,990	712,774	70,107	3,851	10,167
	DCF	**OTB17**	VL2440	24.9	69.3	1,333	234,663	619,509	47,268	4,965	13,106
	DCF	**OTB18**	VL2440	25.1	69.8	1,344	236,465	624,267	44,186	5,352	14,128
	DCF	**OTB19**	VL2440	25.3	67.1	1,291	227,175	599,741	65,142	3,487	9,207
	DCF	**OTB20**	VL2440	27.8	87.8	1,689	297,339	784,976	66,667	4,460	11,775
	DCF	**OTB21**	VL2440	29.3	93.2	1,794	315,665	833,357	57,550	5,485	14,481
		**Mean** *(CI95%)*								**4,243** *(3,805*–*4,680)*	**11,201** *(10,046*–*12,356)*

Vessels are listed according to an ascending order of vessel length overall (LOA). Daily (dFC) and annual fuel consumption (yFC), annual GHG emission (yGHG), annual landings, fuel use intensity (FUI, litres of fuel per ton of landed fish) and carbon footprint (CF, kg of CO2-eq per ton of landed fish) are reported for shrimp fishery (Strait of Sicily) and fisheries targeting mixed demersal species. The data source can be either the current on-site investigation (AUDIT) or the logbooks and landing declarations (DCF). See Table 3 for specifications of the parameters and metrics. Regardless of target species, landings refer to the overall catch (e.g., all landed species). Vessel length segment (VL) is assigned based on LOA (VL0612: vessel between 6 and 12 m; VL1218: vessel between 12 and 18 m; VL1824: vessel between 18 and 24 m; VL2440: vessel between 24 and 40 m). See Supplementary Information for details on the landings by main species.

**Table 6:** Fuel use intensity (FUI) and carbon footprint (CF) in Rapido beam trawl (TBB).

Target species (Area)	Data source	Vessel ID	VL	LOA [m]	hFC [l/h]	dFC [l/day]	yFC [l fuel/year]	yGHG [kg CO2/year]	Landings [kg/year]	FUI [l fuel/t fish]	CF [kg CO2/t fish]
**Sole** (Northern Adriatic)	DCF	**TBB03**	VL1218	14.9	43.8	843	148,415	391,817	37,743	4,066	10,735
	DCF	**TBB04**	VL1824	18.1	67.0	1,289	226,845	598,871	37,830	5,996	15,831
	DCF	**TBB06**	VL2440	24.4	117.3	2,257	397,298	1,048,866	66,911	5,938	15,676
	DCF	**TBB07**	VL2440	24.6	117.4	2,260	397,718	1,049,975	70,125	5,672	14,973
		**Mean** *(CI95%)*								**5,418** *(3,967*–*6,869)*	**14,304** *(10,472*–*18,135)*
**Sole, murex** (Central Adriatic)	DCF	**TBB02**	VL1218	13.1	33.0	636	111,970	295,601	36,520	3,066	8,094
	DCF	**TBB05**	VL1824	21.9	90.3	1,738	305,890	807,551	106,221	2,880	7,603
	AUDIT	**TBB01**	VL2440	25.9	115.6	2,225	391,548	1,033,686	204,133	1,918	5,064
	DCF	**TBB08**	VL2440	26.3	120.4	2,318	407,985	1,077,082	190,539	2,141	5,653
	DCF	**TBB09**	VL2440	26.9	128.4	2,473	435,163	1,148,830	177,037	2,458	6,489
		**Mean** *(CI95%)*								**2,493** *(1,893*–*3,092)*	**6,581** *(4,997*–*8,164)*

Vessels are listed according to an ascending order of vessel length overall (LOA). Daily (dFC) and annual fuel consumption (yFC), annual GHG emission (yGHG), annual landings, fuel use intensity (FUI, litres of fuel per ton of landed fish) and carbon footprint (CF, kg of CO2-eq per ton of landed fish) are reported for common sole fishery (Northern Adriatic) and fishery targeting both common sole and purple dye murex species (Central Adriatic). The data source can be either the current on-site investigation (AUDIT) or the logbooks and landing declarations (DCF). See Table 3 for specifications of the parameters and metrics. Regardless target species, landings refer to the overall catch (e.g., all landed species). Vessel length segment (VL) is assigned based on LOA (VL0612: vessel between 6 and 12 m; VL1218: vessel between 12 and 18 m; VL1824: vessel between 18 and 24 m; VL2440: vessel between 24 and 40 m). See Supplementary Information for details on the landings by main species.

**Table 7:** Fuel use intensity (FUI) and carbon footprint (CF) in midwater pair trawl (PTM).

Target species (Area)	Data source	Vessel ID	VL	LOA [m]	hFC [l/h]	dFC [l/day]	yFC [l fuel/year]	yGHG [kg CO2/year]	Landings [kg/year]	FUI [l fuel/t fish]	CF [kg CO2/t fish]
**Anchovy, sardine** (Northern Adriatic)	DCF	**PTM08**	VL1218	13.7	46.5	558	98,123	259,044	415,869	236	623
	DCF	**PTM09**	VL1218	13.9	41.8	502	88,270	233,033	400,390	220	582
	DCF	**PTM10**	VL1218	15.0	51.3	615	108,299	285,909	263,054	412	1,087
	DCF	**PTM11**	VL1218	16.9	50.9	611	107,582	284,017	257,445	418	1,103
	DCF	**PTM12**	VL1218	17.8	53.7	644	113,424	299,438	324,165	350	924
	DCF	**PTM13**	VL1218	17.8	49.8	598	105,228	277,802	262,077	402	1,060
	DCF	**PTM14**	VL1824	21.3	71.9	863	151,809	400,776	483,360	314	829
	DCF	**PTM15**	VL1824	21.7	72.3	868	152,752	403,264	803,587	190	502
	DCF	**PTM16**	VL1824	21.7	76.4	916	161,276	425,769	859,026	188	496
	DCF	**PTM17**	VL1824	22.0	74.0	888	156,223	412,429	780,261	200	529
	DCF	**PTM18**	VL2440	24.8	88.6	1,063	187,026	493,748	861,166	217	573
	DCF	**PTM19**	VL2440	24.8	90.9	1,091	191,972	506,807	915,795	210	553
		**Mean** *(CI95%)*								**280** *(221*–*339)*	**738** *(583*–*894)*
**Anchovy, sardine** (Central Adriatic)	AUDIT	**PTM04**	VL2440	25.5	84.0	1,008	177,378	468,277	342,862	517	1,366
	AUDIT	**PTM05**	VL2440	25.9	85.7	1,029	181,069	478,022	335,370	540	1,425
	AUDIT	**PTM03**	VL2440	26.5	93.1	1,117	196,656	519,173	365,997	537	1,419
	AUDIT	**PTM07**	VL2440	27.0	97.2	1,167	205,324	542,055	365,947	561	1,481
	DCF	**PTM25**	VL2440	28.4	107.9	1,295	227,920	601,709	366,410	622	1,642
	AUDIT	**PTM01**	VL2440	28.6	105.1	1,261	222,021	586,134	369,704	601	1,585
	AUDIT	**PTM02**	VL2440	29.0	96.2	1,154	203,189	536,420	381,163	533	1,407
	AUDIT	**PTM06**	VL2440	29.0	105.3	1,264	222,396	587,124	360,129	618	1,630
		**Mean** *(CI95%)*								**566** *(532*–*601)*	**1,495** *(1,403*–*1,586)*
**Anchovy, sardine** (Southern Adriatic, Sicily)	DCF	**PTM20**	VL1218	16.1	54.9	659	115,958	306,129	106,168	1,092	2,883
	DCF	**PTM21**	VL1824	19.3	64.6	775	136,360	359,991	112,070	1,217	3,212
	DCF	**PTM22**	VL1824	20.3	61.9	743	130,811	345,341	122,747	1,066	2,813
	DCF	**PTM23**	VL1824	23.6	76.8	921	162,148	428,071	135,292	1,199	3,164
	DCF	**PTM24**	VL2440	26.3	99.5	1,194	210,149	554,794	198,799	1,057	2,791
		**Mean** *(CI95%)*								**1,126** *(1,032*–*1,220)*	**2,973** *(2,724*–*3,221)*

Vessels are listed according to an ascending order of vessel length overall (LOA). Daily (dFC) and annual fuel consumption (yFC), annual GHG emission (yGHG), annual landings, fuel use intensity (FUI, litres of fuel per ton of landed fish) and carbon footprint (CF, kg of CO2-eq per ton of landed fish) are reported for Northern-, Central-, and Southern Adriatic and Sicily. The data source can be either the current on-site investigation (AUDIT) or the logbooks and landing declarations (DCF). See Table 3 for specifications of the parameters and metrics. Landings refer to the catch sum of anchovies and sardines. Vessel length segment (VL) is assigned based on LOA (VL0612: vessel between 6 and 12 m; VL1218: vessel between 12 and 18 m; VL1824: vessel between 18 and 24 m; VL2440: vessel between 24 and 40 m). See Supplementary Information for details on the landings by main species.

According to Table 5, the most energy-intensive métier is the bottom otter trawl targeting shrimps in the Strait of Sicily (OTB03-OTB07). Fuel consumption is estimated at around 11.4 litres per kg caught fish and shrimps. Supplementary Information provides details on the landings by main species. Fisheries targeting mixed demersal species were also relatively energy-intensive. Fuel consumption for this métier was around 4.2 litre per kg of caught fish (Table 5).

Special considerations deserve the analysis of Rapido beam trawl fisheries in the Adriatic Sea (Table 6). Common sole and other flatfish used to be important target species for Rapido beam trawl fisheries. The common sole stock is not yet depleted but faces a growth overfishing observed since 2006^66^. In spite of the high level of fishing mortality, purple dye murex has become an increasingly important bycatch species, especially for Rapido beam trawlers in Central Adriatic, which have smaller, but still significant, fuel use intensity than beam trawlers targeting only common sole in Northern Adriatic: around 2.5 and 5.4 litres of fuel per kg of caught fish and invertebrates, respectively. In effect, the fuel consumptions of these two métiers are comparable, for example, the segment VL2440 has, on average, daily consumption of 2,300 l/day (Table 6). But the bulk of catches yielded by purple dye murex halved FUI when they are caught. Supplementary Information shows that, while purple dye murex yields more than 82 tons per vessel annually, only 7 tons/vessel are landed in Northern Adriatic. Since, in economic terms, common sole used to be the main target species for both métiers, with 25 tons/year per vessel, it is worth underlining that 13.6 litres of fuel (*CI95%: 10.5*–*16.6 l/kg*) are required to obtain a kg of common sole in Adriatic.

Midwater pair trawlers targeting anchovies and sardine (see Supplementary Information for landings by species) are the least energy-intensive métiers (Table 7). Furthermore, in Northern Adriatic, industrial fish meat is not often used directly for human consumption, but instead, large parts of unfilleted fish are processed into feed for farmed tuna. Such large catches in the Northern Adriatic fleet halves FUI to 0.28 l/kg of fish compared to the Central Adriatic (0.57 l/kg), further reducing to a third of that estimated for the Southern Adriatic and Sicily (1.3 l/kg), whereas fuel consumption resulted similar in all fleets. For example, for the segment VL2440 we estimate an even daily fuel consumption of 1,150 l/vessel (*CI95%: 1,084*–*1,215*) (Table 7).

**Fig. 9:**
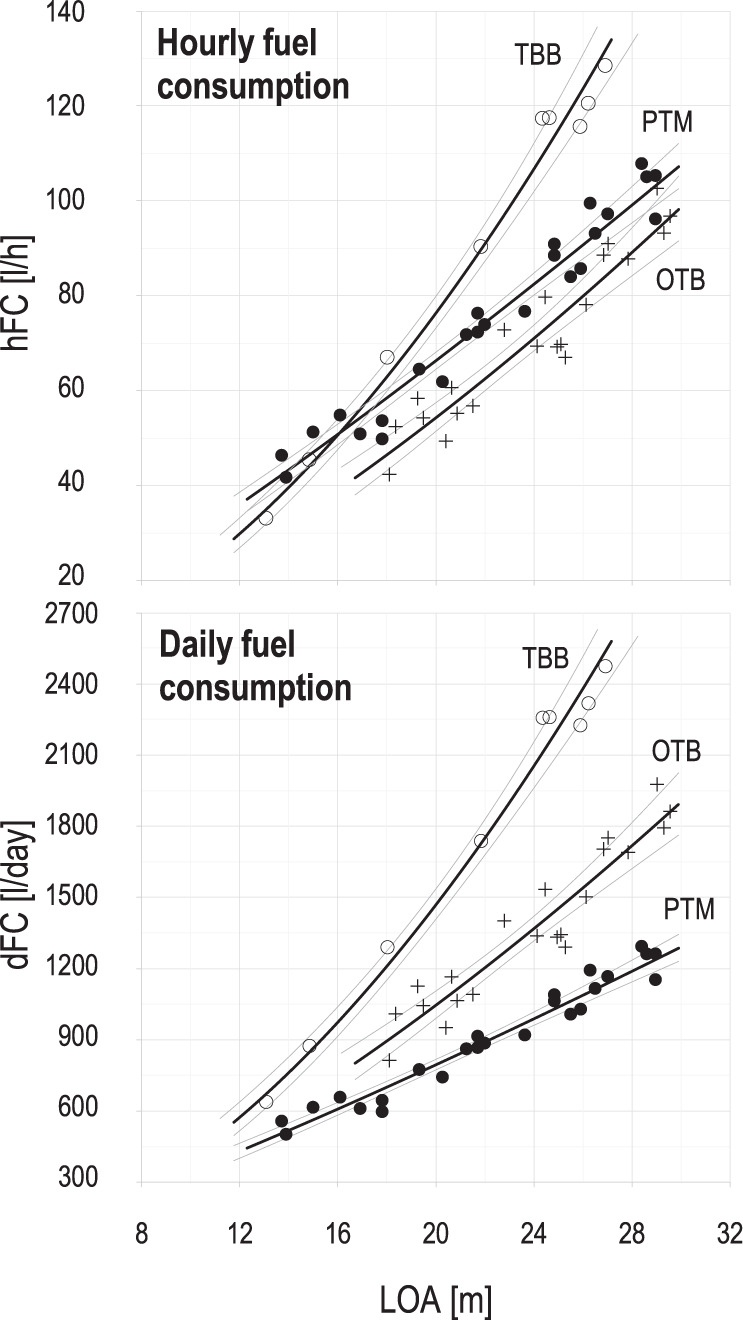
Mean hourly and daily fuel consumption (hFC and dFC, respectively) against vessel length overall (LOA). The linear regression models provide fuel consumption estimates for OTB (+), TBB (○), and PTM (●). The fuel consumption is a weighted average accounting for the relative contribution, or weight, of the steaming and towing working hours in an ordinary week (see Table 2).

The regression model results, developed to infer daily fuel consumption from vessel length, are summarised in Table 8, while the corresponding regression curves are shown in Fig. 9. The mean daily fuel consumptions have been calculated considering 176 days/year at sea and 77 hours/week of fishing activity for OTB and TBB, and 48 hours/week for PTM (see Table 2 for details). Therefore, the model in Table 8 can be used to estimate also the mean hourly fuel consumption for each fishery. The *R-square*, ranging from 0.893 to 0.990, indicates that a good fit to the data was achieved. Notably, for vessels of the same length, an OTB has significantly lower hourly fuel consumption than a PTM (Fig. 9), but in general, the time spent on a daily commercial fishing trip is much higher (e.g., 77 hours per week against 48 for PTM, see Table 2 for details). As such, the daily fuel consumption of an OTB is significantly higher when compared to a PTM of the same LOA.

**Table 8:** Linear regression models to infer daily-fuel consumption, dFC[l/day], from the vessel length overall covariate, LOA[m].

Parameters/vessel type	Daily consumption (dFC)		
	OTB	PTM	TBB
slope, *m*	1.470	1.196	1.838
intercept, *q*	12.811	22.104	5.973
*F*	*158.1*	*475.7*	*666.6*
*d.o.f*	19	23	7
*R-square*	*0.893*	*0.954*	*0.990*

The theoretical LOA-based fuel use models respond to the relationships between daily fuel consumption and vessel length overall (LOA) of the combined analysed dataset (e.g., energy audits and high-resolution logbook dataset). The model coefficient estimates and summary statistics are reported for single boat bottom otter trawlers (OTB), midwater pair trawlers (PTM), and Rapido beam trawlers (TBB). The fuel consumption is a weighted average accounting the relative contribution, or weight, of the steaming and towing working hours in an ordinary week (see Table 2).

General linear model: $FC\left[l/day\right]=q\times LO{A}^{m}$. The mean daily fuel consumptions have been calculated considering 176 days/year at sea and on average 77 hours/week of fishing activity for OTB, TBB and 48 hours/week for PTM (see Table 2 for details). Therefore, the model can be used to estimate also the mean hourly fuel consumption for each vessel type.

Based on FDI aggregated fleet-wide fishing effort and catch data, the regression models reported in Table 8 have been used to calculate fuel use, FUI and CF of the whole three fleets OTB, TBB, and PTM. Larger vessels tend to have higher landings per fishing day, but also higher fuel use (Table 9). Large vessels burn more fuel per unit of effort than small ones. Larger annual landings are hence outbalanced by the higher fuel use of larger vessels, which makes the difference in fuel use per landing between the size segments remarkably small. As confirmed by the present study and Thrane^31^, the indicator ‘litres of fuel per ton of landed fish’, hence carbon footprint, varies according to the fishing gear used, together with the vessel size. Therefore, an energy-efficient solution for one may not be adequate for another vessel.

**Table 9:** Estimated fuel use intensity (FUI) and carbon footprint (CF).

Vessel type	VL	Time at sea	Landings		yFC	yGHG	dFC	FUI	CF
		[days/year]	[t/year]	[kg/boat/year]	[l × 1000 fuel/year]	[t CO2/year]	[l fuel/boat/day]	[l fuel/t fish]	[kg CO2/t fish]
**OTB**	VL1218	157,280	25,272	28,280	98,125 *(35.3%)*	259,049	624 *(494*–*824)*	3,883 *(3,073*–*5,128)*	10,250 *(8,114*–*13,538)*
	VL1824	91,806	24,210	46,412	98,801 *(35.6%)*	260,835	1,076 *(896*–*1,285)*	4,081 *(3,398*–*4,872)*	10,774 *(8,972*–*12,862)*
	VL2440	30,770	9,599	54,905	49,229 *(17.7%)*	129,965	1,600 *(1,368*–*2,184)*	5,129 *(4,384*–*5,499)*	13,539 *(11,575*–*14,518)*
		**279,856**	**59,081**	**37,156**	**246,155** ***(88.7%)***	**649,849**	**880** ***(721***–***1,038)***	**4,085** ***(3,778***–***4,391)***	**10,784** ***(9,974***–***11,593)***
**PTM**	VL1218	4,857	7,950	288,079	2,533 *(0.9%)*	6,687	521 *(432*–*655)*	319 *(264*–*400)*	841 *(697*–*1,057)*
	VL1824	6,178	13,656	389,043	5,024 *(1.8%)*	13,263	813 *(701*–*940)*	368 *(317*–*425)*	971 *(838*–*1,123)*
	VL2440	4,426	13,937	554,193	4,970 *(1.8%)*	13,121	1,123 *(990*–*1,448)*	357 *(314*–*378)*	941 *(830*–*998)*
		**15,461**	**35,543**	**404,603**	**12,527** ***(4.5%)***	**33,071**	**810** ***(698***–***923)***	**349** ***(330***–***369)***	**922** ***(871***–***973)***
**TBB**	VL1218	1,740	355	35,908	1,345 *(0.5%)*	3,551	773 *(575*–*1,091)*	3,789 *(2,819*–*5,348)*	10,004 *(7,443*–*14,118)*
	VL1824	3,678	1,117	53,451	5,617 *(2.0%)*	14,830	1,527 *(1,212*–*1,902)*	5,029 *(3,991*–*6,262)*	13,276 *(10,535*–*16,532)*
	VL2440	4,764	2,587	95,573	11,948 *(4.3%)*	31,543	2,508 *(2,056*–*3,693)*	4,618 *(3,787*–*5,028)*	12,193 *(9,998*–*13,273)*
		10,182	4,059	70,161	18,911 *(6.8%)*	49,924	1,857 *(1,525*–*2,189)*	4,625 *(4,239*–*5,012)*	12,210 *(11,190*–*13,230)*
**Total**	**VL1240**	**305,499**	**98,683**	**56,852**	**277,592**	**732,843**	**909** ***(737***–***1,080)***	**2,895** ***(2,696***–***3,095)***	**7,643** ***(7,116***–***8,170)***

Annual fuel consumption (yFC) and GHG emission (yGHG), fuel use intensity (FUI, litres of fuel per ton of landed fish), and carbon footprint (CF, kg of CO2-eq per ton of landed fish) provided for three major trawl fleets of the Mediterranean: single boat bottom otter trawler (OTB), midwater pair trawler (PTM), and Rapido beam trawler (TBB). For midwater pair trawl (PTM), landings refer to the catch sum of anchovies and sardine, while for single boat bottom otter trawl (OTB) and Rapido beam trawl (TBB) to the overall catch (e.g., all landed species). Information on days at sea and landings are elaborated on complementary data obtained from the Scientific Fisheries Dependent Information (FDI) database. Vessel length segment (VL) is based on LOA (VL1218: vessel between 12 and 18 m; VL1824: vessel between 18 and 24 m; VL2440: vessel between 24 and 40 m). See Table 3 for details of the parameters and metrics.

Note: for dFC, FUI, and CF the figures represent the weighted mean and 95% Confidence Interval (in parenthesis). The weighted average accounts for the relative contribution, or weight, of the fishing days in each vessel length segment (VL).

Similarly, the energy audit, together with the feedback from the shipowner, is the key to determining the suitability of energy-efficient measures onboard. Rising fuel costs have promoted research and development of various energy-saving technologies, but fuel continues to be a major cost and the catching sector remains exposed to progressively increasing fuel price. Increasing fuel price often results in governments establishing fuel subsidies to support the viability of fishing activities^8,26,67,68^ but such subsidies often work against the development of energy-efficient fishing activities. The European Fisheries Fund could be used to facilitate the shift to less fuel-intensive and low-impact fishing methods and gears. In addition, strong consumer demand for fish products with a small carbon footprint could facilitate a shift to ‘green’ products.

### Comparison of the fuel use and carbon footprint with international fisheries

The FUI and the carbon footprint indicators estimated in the current study are consistent with other findings^7,31,48,57–60,69^–^93^, but the trawl fisheries examined here were substantially more fuel-intensive than most fisheries around the world. In detail, Table 10 summarises the figures from the available literature. In general, the relationships found in Italian trawl fisheries between FUI, target species and gear type reflect those found previously in other regions and confirm that on average around 2,0–3.0 litre of fuel is burned per kg of landed fish (e.g., compare Table 9 and Table 10). Furthermore, the pattern of demersal fisheries burning considerably greater amounts of fuel than fisheries targeting pelagic finfish and small pelagics, is validated (Table 10). However, it is worth remarking that the fish caught with pelagic trawls are made up of sardine and anchovies, which are typically lower priced than the other catch the vessels obtain with bottom trawl gears.

**Table 10:** Review of published studies on fuel use intensity (FUI) in trawl fisheries.

Target species/Gears	FUI [l/t]					
	No.	Min	Max	Mean	CI95%	References
**Small pelagics**						
Midwater otter trawls	26	81	1,097	**360**	*(243*–*478)*	^59,69–74^
**Demersal species**						
Beam trawls	2	980	2,610	**1,795**	*(0*–*12,151)*	^31,75^
Bottom otter trawls	139	326	17,560	**2,970**	*(2,441*–*3,499)*	^7,31,57,59,60,70,71,75–91^
Midwater otter trawls	10	377	2,342	**1,114**	*(704*–*1,524)*	^69,70,80^
Overall				**2,832**	*(2,339-3,325)*	
**All trawl gears**				**2,469**	*(2,029-2,909)*	

Number of records found in the available references, with the minimum, maximum, and mean values reported together with the calculated 95% Confidence Intervals. The fishing gears are separated in trawls targeting small pelagics and demersal species.

Parker *et al*.^48^ estimate that the world’s fishing fleets in 2011 burned 40 billion litres of fuel and emitted 179 million tonnes of CO2-equivalent to the atmosphere, or 2.2 kg CO2-eq per kg of landed fish and invertebrates. According to the authors, fuel-related GHG emissions were calculated using 3.1 kg CO2-eq per litre, to account for direct emissions from burning fuel as well as emissions from upstream mining, processing and transport of fuel^48^. Assuming a total direct emission from burning fuel of 2.64 kg CO2-eq per litre of fuel, based on the chemical content of marine fuels^42,43^, their estimated harvest source of emission is quantifiable at around 1.9 kg CO2-eq per kg of landed fish and invertebrates. Which, in other terms, can be expressed as a globally averaged FUI of all fisheries in 710 litres of fuel per ton of landed fish.

All but two pelagic métiers assessed here have a higher FUI than this global average (Tables 5–7). This is due to the fisheries targeting fuel-intensive shrimps and flatfish. However, Italian fisheries tend to demand more energy inputs even when compared based on similar species and gears. For example, in a study by Parker *et al*.^59^, the small-pelagics trawl fisheries burned, on average, 92–164 litres per ton of fish during the harvesting activity, against 280–1,126 l/t of the current study (Table 7). While the bottom otter trawl fisheries ranged between 907–1,091 and 1,503–9,685 l/t^59^ litre per ton of landed finfish and prawn, respectively. Likewise, Basurko *et al*.^57^ assessed for a Spanish otter bottom trawler an FUI of 1,646 litres of fuel per ton of landed fish, and Schau *et al*.^69^ quantified an FUI of 105 and 1,209 l/t for a Norwegian shrimp trawl and mid-water blue whiting fisheries, respectively.

In the current study, bottom trawlers targeting mixed demersal species and shrimps confirm this general tendency with an FUI ranging between 4,243 and 11,379 l/t, respectively (Table 5), being more ‘fuel intensive’ than pelagic trawlers. No specific references were found for Rapido beam trawler, which evidently is a fishery monitored for the first time in the present study. Other experiments^31,75^, on fuel consumption patterns by gear types report that beam trawlers targeting flatfish generally require higher amounts of fuel (approximately 980–2,610 litre of fuel per ton of fish) than bottom otter trawls of the same vessel segment (Table 10). The results obtained in this study confirm these rates (2,493–5,418 l/t, see Table 6) and may be used as a benchmark for this fishing gear. However, it must be noted that each vessel behaves differently, despite operating with similar gear. Operational techniques and the distances between fishing grounds and fishing ports, as well as vessel and gear design and size will all affect the amount of fuel consumed. There are also substantial differences in fuel use intensity yielded by the target and bycatch availability, such as the differences between the Northern and Central Adriatic Rapido beam trawlers.

## Usage Notes

The datasets are available for three main Mediterranean trawl fisheries: single boat bottom otter trawlers (OTB), midwater pair trawlers (PTM), and Rapido beam trawlers (TBB). The data analysis implied either reading flat files or bulk-importing data into a dedicated database while ensuring that relevant fields are well indexed. The descriptive fields inherent to the database will enable the sub-setting of the data, which is helpful for further subsequent analysis.

## Supplementary information

Supplementary Information - Energy audit and carbon footprint in trawl fisheries
