# Supplementary material for: Energy audit and carbon footprint in trawl fisheries
Source: Sci Data. 2022 Jul 20;9:428. doi: 10.1038/s41597-022-01478-0 (PMC9300640; doi:10.1038/s41597-022-01478-0)
Supplement: Supplementary file 1 — Supplementary Information - Energy audit and carbon footprint in trawl fisheries [file 41597_2022_1478_MOESM1_ESM.docx]

# Supplementary Information File

# Energy audit and carbon footprint in trawl fisheries

Antonello Sala ^1,*^, Dimitrios Damalas ^2^, Lucio Labanchi ^3^, Jann Martinsohn ^4^, Fabrizio Moro ^1^, Rosaria Sabatella ^5^, Emilio Notti ^1^

^1^ National Research Council, Institute of Marine Biological Resources and Biotechnologies (CNR-IRBIM), Ancona, Italy. ^2^ Institute of Marine Biological Resources and Inland Waters, Hellenic Centre for Marine Research, Heraklion, Greece. ^3^ MARBLY scarl, Salerno, Italy. ^4^ European Commission, Joint Research Centre (JRC), Ispra (VA), Italy. ^5^ NISEA, Fisheries and Aquaculture Economic Research, Salerno, Italy.

*Corresponding author email: [antonello.sala@cnr.it](mailto:antonello.sala@cnr.it)

## Supplementary Table 1. Total annual landings (kg/year) by single boat shrimp otter trawlers (OTB) in the Strait of Sicily (Italy). Mean and 95% Confidence Interval *(CI95%)* of the overall yield (kg of landed fish by year) and by main species landed by each trawler are reported. Scientific, English name, and 3-alpha code of the species (3ACODE) are based on the FAO Aquatic Science and Fisheries Information System (ASFIS)^61^.

| **Species** |  |  | **Shrimp otter trawlers (Strait of Sicily)** | | | |  |  |
| --- | --- | --- | --- | --- | --- | --- | --- | --- |
| 3ACODE | *Scientific name* | English name | **OTB03** | **OTB04** | **OTB05** | **OTB06** | **OTB07** | **Mean *(CI95%)*** |
| ARS | *Aristaeomorpha foliacea* | Giant red shrimp | 10,686 | 6,751 | 17,427 | 20,323 | 11,040 | **13,245** *(6,411-20,079)* |
| ARA | *Aristeus antennatus* | Blue and red shrimp | 6,423 | 7,488 | 6,894 | 7,914 | 6,557 | **7,055** *(6,270-7,840)* |
| DPS | *Parapenaeus longirostris* | Deep-water rose shrimp | 3,977 | 1,831 | 918 | 750 | 920 | **1,679** *(0-3,359)* |
| MUX | *Mullus spp* | Red mullets | 906 | 1,549 | 0 | 0 | 1,491 | **789** *(0-1,737)* |
| NEP | *Nephrops norvegicus* | Norway lobster | 914 | 306 | 460 | 297 | 1,796 | **755** *(0-1,541)* |
| HKE | *Merluccius merluccius* | European hake | 171 | 521 | 412 | 453 | 1,966 | **705** *(0-1,595)* |
| **Total landing** | | | **23,283** | **27,985** | **26,791** | **30,778** | **27,300** | **27,227** *(23,888-30,566)* |

## Supplementary Table 2. Total annual landings (kg/year) by single boat bottom otter trawlers (OTB) in the Italian seas. Mean and 95% Confidence Interval *(CI95%)* of the overall yield (kg of landed fish by year) and by main species landed by each trawler are reported. Scientific, English name, and 3-alpha code of the species (3ACODE) are based on the FAO Aquatic Science and Fisheries Information System (ASFIS)^61^.

| **Species** | | | **Single boat bottom otter trawlers (all Italian seas)** | | | | | | | | | | | | | | | | |
| --- | --- | --- | --- | --- | --- | --- | --- | --- | --- | --- | --- | --- | --- | --- | --- | --- | --- | --- | --- |
| 3ACODE | *Scientific name* | English name | **OTB08** | **OTB09** | **OTB10** | **OTB11** | **OTB12** | **OTB13** | **OTB14** | **OTB01** | **OTB02** | **OTB15** | **OTB16** | **OTB17** | **OTB18** | **OTB19** | **OTB20** | **OTB21** | **Mean *(CI95%)*** |
| DPS | *Parapenaeus longirostris* | Deep-water rose shrimp | 4,819 | 1,292 | 18,763 | 12,895 | 3,130 | 13,612 | 3,652 | 585 | 649 | 10,033 | 28,947 | 8,475 | 0 | 22,275 | 23,750 | 1,671 | **9,659**  *(4,636-14,683)* |
| HKE | *Merluccius merluccius* | European hake | 1,957 | 7,279 | 4,362 | 2,239 | 2,976 | 6,497 | 2,668 | 7,463 | 6,864 | 12,926 | 12,805 | 2,188 | 5,199 | 7,600 | 11,768 | 6,472 | **6,329**  *(4,374-8,284)* |
| MUX | *Mullus spp* | Red mullets | 554 | 9,335 | 249 | 4,589 | 2,014 | 5,919 | 3,507 | 12,148 | 18,784 | 7,978 | 3,213 | 5,732 | 3,311 | 3,019 | 3,002 | 1,667 | **5,314**  *(2,750-7,878)* |
| MTS | *Squilla mantis* | Spottail mantis squillid | 280 | 10,056 | 0 | 777 | 1,651 | 3,609 | 2,068 | 4,801 | 9,623 | 3,135 | 839 | 0 | 2,450 | 0 | 1,571 | 0 | **2,554**  *(857-4,251)* |
| OMZ | *Ommastrephidae* | Ommastrephidae squids | 1,051 | 1,275 | 2,028 | 3,029 | 1,107 | 2,347 | 1,316 | 2,323 | 2,266 | 5,338 | 3,227 | 2,167 | 1,002 | 3,544 | 4,279 | 1,771 | **2,379**  *(1,719-3,040)* |
| OCM | *Eledone spp* | Horned & musky octopuses | 808 | 1,008 | 66 | 3,468 | 3,720 | 4,522 | 4,080 | 0 | 0 | 3,884 | 5,068 | 379 | 772 | 0 | 5,904 | 171 | **2,116**  *(969-3,262)* |
| NEP | *Nephrops norvegicus* | Norway lobster | 0 | 431 | 114 | 1,325 | 18 | 86 | 294 | 3,065 | 2,083 | 7,036 | 3,714 | 840 | 0 | 8,898 | 4,406 | 300 | **2,038**  *(582-3,494)* |
| ARS | *Aristaeomorpha foliacea* | Giant red shrimp | 5,497 | - | 1,365 | 247 | 0 | 0 | 1,383 | - | - | - | 0 | 7,199 | 404 | 0 | 140 | 6,968 | **1,934**  *(119-3,748)* |
| WHG | *Merlangius merlangus* | Whiting | - | 2,493 | - | - | - | - | - | 2,756 | 234 | 1,642 | - | - | - | - | - | - | **1,781**  *(0-3,589)* |
| MNZ | *Lophius spp* | Monkfishes | 254 | 2,743 | 497 | 680 | 472 | 630 | 191 | 3,531 | 2,571 | 5,237 | 1,155 | 4,600 | 178 | 1,280 | 1,280 | 223 | **1,595**  *(719-2,472)* |
| POD | *Trisopterus minutus* | Poor cod | - | 1,324 | - | 1,094 | - | - | 1,556 | 1,706 | 2,367 | 905 | - | - | - | - | - | - | **1,492**  *(947-2,036)* |
| ARA | *Aristeus antennatus* | Blue & red shrimp | 6,932 | - | 0 | 189 | 0 | 0 | 0 | - | - | - | - | 0 | 0 | 0 | - | 7,003 | **1,412**  *(0-3,507)* |
| TGS | *Penaeus kerathurus* | Caramote prawn | - | 3,417 | - | 21 | - | - | 1,475 | 1,171 | 2,906 | 1,291 | 343 | - | - | - | 213 | - | **1,355**  *(315-2,394)* |
| IAX | *Sepia spp* | Cuttlefishes | 462 | 2,486 | 914 | 301 | 1,470 | 2,758 | 1,018 | 2,295 | 3,693 | 907 | 1,733 | 771 | 209 | 0 | 1,917 | 266 | **1,325**  *(755-1,895)* |
| GUX | *Triglidae* | Gurnards | 0 | 1,738 | 40 | 1,213 | 1,207 | 2,233 | 168 | 4,307 | 2,598 | 1,855 | 1,334 | 0 | 1,338 | 0 | 1,145 | 1,122 | **1,269**  *(652-1,885)* |
| OCC | *Octopus vulgaris* | Common octopus | 611 | 205 | 175 | 444 | 417 | 4,329 | 2,170 | 1,091 | 2,367 | 185 | 583 | 1,013 | 1,681 | 79 | 533 | 859 | **1,046**  *(450-1,643)* |
| RAJ | *Rajidae* | Rays & skates | 0 | 796 | 1,169 | 874 | 844 | 1,559 | 110 | 814 | 1,168 | 302 | 247 | 349 | 1,127 | 4,793 | 247 | 1,061 | **966**  *(371-1,561)* |
| JAX | *Trachurus spp* | Jack & horse mackerels | 569 | 231 | 529 | 2,078 | 626 | 577 | 600 | 839 | 138 | 2,606 | 120 | 1,814 | 2,845 | 0 | 594 | 1,258 | **964**  *(487-1,441)* |
| MAZ | *Scomber spp* | Scomber mackerels | - | 1,232 | - | 227 | 245 | - | - | 418 | 1,605 | 2,260 | 536 | 40 | - | - | 367 | 24 | **695**  *(158-1,233)* |
| SQC | *Loligo spp* | Common squids | 717 | 530 | 710 | 103 | 1,272 | 0 | 532 | 1,271 | 1,052 | 133 | 268 | 1,370 | 1,157 | 0 | 129 | 1,135 | **649**  *(380-917)* |
| **Total landing** | |  | **31,797** | **56,951** | **50,511** | **42,432** | **31,682** | **51,690** | **36,638** | **58,128** | **65,803** | **78,807** | **70,107** | **47,268** | **44,186** | **65,142** | **66,667** | **57,550** | **53,460**  *(46,028-60,892)* |

Note: if a species is not available in the area (-) it was not considered in the mean calculation.

## Supplementary Table 3. Total annual landings (kg/year) by Rapido beam trawlers (TBB) harvesting either common sole (Northern Adriatic) or common sole and murex (Central Adriatic). Mean and 95% Confidence Interval *(CI95%)* of the overall yield (kg of landed fish by year) and by main species landed by each trawler are reported. Scientific, English name, and 3-alpha code of the species (3ACODE) are based on the FAO Aquatic Science and Fisheries Information System (ASFIS)^61^.

| **Species** |  |  | **Rapido beam trawlers targeting sole (Northern Adriatic)** | | | | | |
| --- | --- | --- | --- | --- | --- | --- | --- | --- |
| 3ACODE | *Scientific name* | English name | **TBB03** | **TBB04** | **TBB06** | **TBB07** |  | **Mean** *(CI95%)* |
| SOL | *Solea solea* | Common sole | 14,245 | 12,278 | 33,485 | 26,410 |  | **21,605** *(5,551-37,659)* |
| IAX | *Sepia spp* | Cuttlefishes | 3,875 | 6,113 | 6,334 | 10,109 |  | **6,608** *(2,494-10,721)* |
| BOY | *Bolinus brandaris* | Purple dye murex | 8,179 | 2,855 | 7,741 | 6,671 |  | **6,361** *(2,508-10,215)* |
| SCX | *Pectinidae* | Scallops | 3,013 | 9,365 | 4,419 | 400 |  | **4,299** *(0-10,291)* |
| MTS | *Squilla mantis* | Spottail mantis squillid | 1,485 | 441 | 3,690 | 4,364 |  | **2,495** *(0-5,424)* |
| OCC | *Octopus vulgaris* | Common octopus | 26 | 27 | 2,494 | 3,505 |  | **1,513** *(0-4,322)* |
| SCS | *Scorpaena spp* | Scorpionfishes, rockfishes | 562 | 998 | 2,033 | 1,805 |  | **1,349** *(255-2,444)* |
| HKE | *Merluccius merluccius* | European hake | 606 | 285 | 1,321 | 2,183 |  | **1,099** *(0-2,439)* |
| TGS | *Penaeus kerathurus* | Caramote prawn | 461 | 820 | 904 | 1,327 |  | **878** *(312-1,444)* |
| GUX | *Triglidae* | Gurnards | 379 | 789 | 1,007 | 1,231 |  | **851** *(274-1,429)* |
| **Total landing** | |  | **37,743** | **37,830** | **66,911** | **70,125** |  | **53,152** *(24,842-81,462)* |
| **Species** |  |  | **Rapido beam trawlers targeting sole and murex (Central Adriatic)** | | | | | |
| 3ACODE | *Scientific name* | English name | **TBB02** | **TBB05** | **TBB01** | **TBB08** | **TBB09** | **Mean** *(CI95%)* |
| BOY | *Bolinus brandaris* | Purple dye murex | 6,101 | 47,023 | 126,304 | 112,409 | 116,237 | **81,615** *(16,374-146,855)* |
| SOL | *Solea solea* | Common sole | 17,688 | 29,663 | 39,050 | 33,581 | 25,973 | **29,191** *(19,193-39,190)* |
| MTS | *Squilla mantis* | Spottail mantis squillid | 5,427 | 7,402 | 11,673 | 16,961 | 11,028 | **10,498** *(4,989-16,007)* |
| IAX | *Sepia spp* | Cuttlefishes | 939 | 5,778 | 5,042 | 4,172 | 4,080 | **4,002** *(1,708-6,296)* |
| TGS | *Penaeus kerathurus* | Caramote prawn | 792 | 3,157 | 2,699 | 5,204 | 4,590 | **3,288** *(1,141-5,435)* |
| GUX | *Triglidae* | Gurnards | 411 | 3,236 | 4,833 | 2,900 | 2,320 | **2,740** *(752-4,728)* |
| GOB | *Gobius spp* | Atlantic gobies | 1,496 | 1,178 | 3,631 | 2,286 | 2,293 | **2,177** *(998-3,355)* |
| MUX | *Mullus spp* | Red mullets | 2,053 | 791 | 2,116 | 2,007 | 1,654 | **1,724** *(1,039-2,409)* |
| HKE | *Merluccius merluccius* | European hake | 59 | 1,979 | 1,130 | 1,439 | 1,267 | **1,175** *(303-2,047)* |
| OYF | *Ostrea edulis* | European flat oyster | 0 | 0 | 4,085 | 976 | 563 | **1,125** *(0-3,242)* |
| **Total landing** | |  | **36,520** | **106,221** | **204,133** | **190,539** | **177,037** | **142,890** *(55,429-230,351)* |

Note: if a species is not available in the area (-) it was not considered in the mean calculation.

## Supplementary Table 4. Total annual landings (kg/year) by midwater pair trawlers (PTM) in three main fishing areas: Norther-, Central-, and Southern Adriatic and Sicily. Mean and 95% Confidence Interval *(CI95%)* of the overall yield (kg of landed fish by year) and by main species landed by each trawler are reported. Scientific, English name and 3-alpha code of the species (3ACODE) are based on the FAO Aquatic Science and Fisheries Information System (ASFIS)^61^.

| **Vessel / species** | ***Engraulis encrasicolus*** | ***Sardina pilchardus*** | **Total landing**  **[kg/year]** |
| --- | --- | --- | --- |
|  | **European anchovy** | **European pilchard** |  |
|  | **ANE [kg/year]** | **PIL [kg/year]** |  |
| **Midwater pair trawlers (Northern Adriatic)** | | | |
| PTM08 | 154,741 | 261,127 | **415,869** |
| PTM09 | 219,869 | 180,521 | **400,390** |
| PTM10 | 172,053 | 91,001 | **263,054** |
| PTM11 | 179,958 | 77,487 | **257,445** |
| PTM12 | 221,792 | 102,373 | **324,165** |
| PTM13 | 172,405 | 89,672 | **262,077** |
| PTM14 | 124,642 | 358,719 | **483,360** |
| PTM15 | 193,869 | 609,718 | **803,587** |
| PTM16 | 196,025 | 663,001 | **859,026** |
| PTM17 | 293,987 | 486,274 | **780,261** |
| PTM18 | 258,748 | 602,418 | **861,166** |
| PTM19 | 268,259 | 647,535 | **915,795** |
| **Mean** *(CI95%)* | **204,696** *(173,058-236,334)* | **347,487** *(193,837-501,138)* | **552,183** *(381,907-722,459)* |
| **Midwater pair trawlers (Central Adriatic)** | | | |
| PTM04 | 216,354 | 126,508 | **342,862** |
| PTM05 | 247,433 | 87,937 | **335,370** |
| PTM03 | 215,068 | 150,929 | **365,997** |
| PTM07 | 222,028 | 143,920 | **365,947** |
| PTM25 | 299,798 | 66,611 | **366,410** |
| PTM01 | 243,775 | 125,928 | **369,704** |
| PTM02 | 291,209 | 89,954 | **381,163** |
| PTM06 | 239,289 | 120,840 | **360,129** |
| **Mean** *(CI95%)* | **246,869** *(219,697-274,041)* | **114,078** *(89,384-138,773)* | **360,948** *(348,517-373,379)* |
| **Midwater pair trawlers (Southern Adriatic, Sicily)** | | | |
| PTM20 | 81,993 | 24,175 | **106,168** |
| PTM21 | 93,383 | 18,687 | **112,070** |
| PTM22 | 96,554 | 26,193 | **122,747** |
| PTM23 | 100,542 | 34,750 | **135,292** |
| PTM24 | 175,648 | 23,151 | **198,799** |
| **Mean** *(CI95%)* | **109,624** *(63,001-156,247)* | **25,391** *(18,054-32,729)* | **135,015** *(88,650-181,381)* |
